# Event-related potentials reflecting smoking cue reactivity and cognitive control as predictors of smoking relapse and resumption

**DOI:** 10.1007/s00213-016-4332-8

**Published:** 2016-06-08

**Authors:** Maartje Luijten, Marloes Kleinjan, Ingmar H. A. Franken

**Affiliations:** Behavioural Science Institute, Radboud University, P.O. Box 9104, 6500 HE Nijmegen, The Netherlands; Institute of Psychology, Erasmus University Rotterdam, P.O. Box 1738, 3000 DR Rotterdam, The Netherlands; Trimbos Institute (Netherlands Institute of Mental Health and Addiction), P.O. Box 725, 3500 AS Utrecht, The Netherlands

**Keywords:** Smoking, Event-related potentials, Smoking cue reactivity, Inhibitory control, Error processing, Smoking cessation, Relapse, Addiction, Substance use

## Abstract

**Rationale:**

Given that most attempts to quit smoking fail, it is critical to increase knowledge about the mechanisms involved in smoking relapse and resumption (i.e., the increase in smoking over time after a quit attempt). Neurocognitive measures, such as event-related potentials (ERPs), may provide novel insights into smoking relapse and resumption.

**Objectives:**

The objective of the present study is to investigate the association between smoking relapse and resumption and ERPs reflecting smoking cue reactivity (i.e., P300, LPP), inhibitory control (i.e., N2, P3), and error processing (i.e., error-related negativity (ERN), Pe).

**Methods:**

Seventy-two smokers viewed smoking and neutral pictures and performed a Go-NoGo and an Eriksen Flanker task, while ERPs were measured using electroencephalography. All smokers started a quit attempt in the week following the laboratory visit. Smoking behavior after the quit attempt was measured at 4, 8, and 12 weeks. Both relapse (i.e., 7-day point prevalence at 12 weeks) and smoking resumption (i.e., the number of cigarettes a day at 4, 8, and 12 weeks) were used as outcome measures.

**Results:**

Logistic regression analyses showed that smaller P3 amplitudes, reflecting brain activation associated with inhibitory control, are related to an increased relapse risk. Latent growth curve analyses showed that reduced post-error slowing, the main behavioral measure reflecting error processing, is associated with stronger smoking resumption. ERPs reflecting smoking cue reactivity were unrelated to smoking relapse or resumption.

**Conclusions:**

The finding that smaller inhibitory control-related P3 amplitudes are associated with increased relapse risks suggests that strategies to increase inhibitory control in smokers are worth further investigation in the search for more effective smoking cessation interventions.

**Electronic supplementary material:**

The online version of this article (doi:10.1007/s00213-016-4332-8) contains supplementary material, which is available to authorized users.

## Introduction

Given the serious health risks associated with smoking as well as the high worldwide smoking prevalence (US Department of Health and Human Services 2014), it is critical to increase successful smoking cessation. About 88–95 % of quitters smoke again in the year following a quit attempt (International Tobacco Control Policy Evaluation Project 2011), and even the most effective interventions have limited effects on long-term abstinence (Hajek et al. 2013). In this context, identification of factors predicting relapse into smoking is crucial to identify further treatment targets. While smoking characteristics such as nicotine dependence levels and craving have previously been associated with smoking relapse (Ferguson and Shiffman 2009; Vangeli et al. 2011; however, see Wray et al. 2013 for inconsistent results regarding craving), present addiction models emphasize the role of neurocognitive factors in addiction such as strong reactivity to substance-related cues and impaired cognitive control (Field and Cox 2008; Goldstein and Volkow 2011; Luijten et al. 2014). Given that the direct relation between these neurocognitive factors and smoking cessation has hardly been investigated, the current study examined the association between smoking relapse and resumption and neurocognitive measures reflecting smoking cue reactivity and cognitive control.

Various addiction models suggest that the interplay between motivational/reward-related processes and control-related processes underlies the continuation of addictive behavior (Field and Cox 2008; Goldstein and Volkow 2011; Wiers et al. 2007). In line with these models, both functional magnetic resonance imaging (fMRI) and electroencephalography (EEG) studies have consistently shown smoking-cue reactivity in smokers, i.e., enhanced processing of smoking-related cues in motivational- and reward-related brain regions, as well as enlarged event-related potentials (ERPs) reflecting attentional processing of smoking cues (for meta-analyses, see Engelmann et al. 2012; Littel et al. 2012). Additionally, it has been shown that processes related to cognitive control, such as inhibitory control and error processing, are reduced in smokers (for review and meta-analysis, see Luijten et al. 2014; Smith et al. 2014). Reduced inhibitory control is defined by the inability to implement the inhibition of inappropriate behavior, while error processing refers to the monitoring of performance errors, which may be associated with future mistakes (Ridderinkhof et al. 2004). While there is ample evidence from cross-sectional studies that smoking cue reactivity, inhibitory control, and error processing are involved in smoking behavior, the prospective association of these processes with smoking cessation is less clear (Stevens et al. 2014). Smoking cue reactivity, or more specifically attentional bias (i.e., the automatic tendency of smokers to direct attention to smoking-related cues), has been found to be associated with smoking relapse in previous studies (Janes et al. 2010; Powell et al. 2010; Waters et al. 2003b). fMRI studies further revealed that brain activation evoked by smoking cues in motivational circuits is related to a larger risk of smoking relapse (Janes et al. 2010; Versace et al. 2014). However, not all studies showed the expected association between attentional bias or brain responses to smoking cues and smoking cessation outcomes (Versace et al. 2012; Waters et al. 2003a). Current findings regarding inhibitory control are also inconclusive. Reduced inhibitory control, as measured with different behavioral measures, was associated with a higher chance of smoking relapse during unaided cessation (Powell et al. 2010) and with lower quit rates in a treatment program for adolescent smokers (Krishnan-Sarin et al. 2007). However, Sheffer et al. (2012) did not find an association between inhibitory control and smoking cessation in a group of lower socioeconomic smokers. Studies investigating the link between brain activation associated with inhibitory control and smoking cessation, or substance use relapse in general, are currently lacking. We also could not identify any study that investigated the link between error processing and smoking cessation. Results in cocaine users, however, suggest that error processing may be valuable in predicting relapse into substance use. Reduced error-related negativity (ERN), i.e., a response-locked ERP that is strongly related to performance monitoring, and reduced error-related ACC activation measured with fMRI were both found to be predictive of increased cocaine use and cocaine relapse after treatment (Luo et al. 2013; Marhe et al. 2013).

Given the inconclusive results in behavioral studies and the paucity of studies investigating the link between neural correlates of smoking cue reactivity, inhibitory control, error processing, and smoking cessation, the current study investigated whether behavioral and/or neural measures of these addiction-relevant processes are associated with smoking relapse and resumption. In order to do so, participants performed a cue reactivity, inhibitory control, and error-processing task, while ERPs and behavioral performance were measured. Both task performance and ERPs were included in the analyses to predict relapse and smoking resumption, i.e., the increase in smoking over time after a quit attempt. The cognitive neuroscience literature has clearly identified the contribution of different ERPs to cue reactivity, inhibitory control, and error processing. The P300 and late positive potential (LPP) are two ERPs associated with smoking cue reactivity representing deployment of attentional processing to salient stimuli and the continuation of attentional processing to facilitate memory storage (Hajcak and Olvet 2008; Koenig and Mecklinger 2008; Littel et al. 2012; Polich and Kok 1995). The P300 is a large positive reflection that is maximal approximately 300–500 ms after stimulus presentation with a medial central and parietal distribution. The P300 is followed by the LPP and is a continuation of the positive reflection for longer time periods. The N2 and P3 are the most important ERPs associated with inhibitory control arising 200–300 and 300–500 ms at frontocentral and parietal-central locations, respectively (Band and Van Boxtel 1999; Enriquez-Geppert et al. 2010; Falkenstein 2006; Kaiser et al. 2006; Luijten et al. 2014; Nieuwenhuis et al. 2003). More specifically, the N2 is seen as an index for early cognitive processes, which has been particularly associated with conflict detection, an important aspect of inhibitory control. The P3 reflects a later stage of the inhibitory process closely related to the actual inhibition of the motor system in the premotor cortex. With regard to error processing, it has been found that performance errors strongly evoke an initial ERN and a subsequent Pe wave. The ERN is maximal at 50–80 ms after an incorrect response and arises mostly from the anterior cingulate cortex. Pe waves generally have a more central-parietal distribution and emerge 200–400 ms after an incorrect response. The ERN and the Pe are, respectively, interpreted as neural measures of fast automatic error detection and the more elaborative, conscious processing of errors (Bernstein et al. 1995; Luijten et al. 2014; Overbeek et al. 2005; Ridderinkhof et al. 2009; Wessel et al. 2011). Previous cross-sectional studies have shown deficits in smokers for all these ERP measures compared to non-smoking controls. Generally, P300 and LPP amplitudes for smoking cues are enlarged in smokers (for meta-analysis, see Littel et al. 2012) and the ERPs representing cognitive control (i.e., the N2, P3, ERN, and Pe) seem to be reduced in smokers (for review, see Luijten et al. 2014).

To summarize, the main aim of the current study is to predict smoking relapse and resumption by means of behavioral indices and ERPs associated with smoking cue reactivity, inhibitory control, and error processing. It is expected that increased processing of smoking cues (i.e., larger P300 and LPP amplitudes), as well as reduced inhibitory control and error processing (i.e., lower inhibitory control accuracy and post-error slowing and smaller N2, P3, ERN, and Pe amplitudes), is associated with a higher chance of relapse and increased smoking over time after the quit attempt.

## Materials and methods

### Participants and procedures

Seventy-two smokers were recruited from the general population through (online) media advertisements and counseling programs for smoking cessation in hospitals. Inclusion criteria were (a) smoking at least weekly, (b) having the intention to quit smoking, and (c) being older than 18. Exclusion criteria were self-reported (a) current mental illness, (b) current or past substance dependence other than nicotine, (c) current use of psychoactive medication other than medication for smoking cessation, and (d) current or past neurological problems. Five participants were excluded from all analyses because of a brain stroke in the past (*n* = 1), previous substance dependence other than nicotine (*n* = 1), current psychosocial treatment (*n* = 1), current use of antidepressants (*n* = 1), and problems with data acquisition for all tasks (*n* = 1). One more participant was excluded for smoking cue reactivity analyses because of problems with data acquisition for this task specifically. Another participant was excluded for analyses regarding inhibitory control as measured with the Go-NoGo task because of missing data for this task. Seven participants were excluded from analyses related to error processing because of less than seven artifact-free ERP epochs in at least one of the task conditions of the Eriksen Flanker task (*n* = 5) (Olvet and Hajcak 2009) or because of problems with data acquisition for the Eriksen Flanker task specifically (*n* = 2). In total, 66 participants were included in analyses related to smoking cue reactivity and inhibitory control, and 60 participants were included in analyses related to error processing.

Detailed participant characteristics are displayed in Table 1. All EEG data was acquired in the week prior to the quit date. Participants were free to use any type of support during the quit attempt (for details on types of support, see Table 1). Those participants who used varenicline (16 %) or bupropion (3 %) were on medication at the time of testing. Participants were asked to abstain from smoking for 1 h before testing. This short period of smoking, abstinence was introduced to reduce the acute effects of nicotine on ERP amplitudes (Houlihan et al. 1996, 2001) without introducing withdrawal effects. Upon arrival, a CO breath sample (Micro+ Smokerlyzer (Bedfont Scientific Ltd., Rochester, UK) was taken and questionnaires were completed. Subsequently, the participants were seated in a comfortable EEG chair in a light- and sound-attenuated room. Electrodes were attached and task instructions were explained. Participants performed three tasks in a fixed order (i.e., Eriksen Flanker task, Go-NoGo task, and smoking cue reactivity, respectively) while EEG was recorded. Follow-up data was obtained via telephone interviews at 4, 8, and 12 weeks after the quit date. Participants received €20 as a compensation for study participation. The study was conducted in accordance with the Declaration of Helsinki, and all participants provided written informed consent. The medical ethics committee for scientific research in the Rotterdam area approved the study, and all study procedures were conducted in accordance with the Declaration of Helsinki.

**Table 1:** Demographics

	Relapsers (*N* = 37)^a^			Non-relapsers (*N* = 25)^a^				
	Mean	SD	Range	Mean	SD	Range	*t*/*X2*	*p*
Gender (% male)	38 %			64 %			4.09	0.043*
Education							3.37	0.185
% Low	41 %			20 %				
% Medium	32 %			52 %				
% High	27 %			28 %				
Age	39.51	16.07	18–70	46.56	16.12	16–68	−1.69	0.096
FTND	3.70	2.15	0–7	3.84	2.41	0–9	−0.24	0.815
AUDIT	6.57	3.81	1–17	5.91	5.65	1–23	0.53	0.599
QSU—baseline	23.89	11.58	10–56	21.28	11.38	10–51	0.88	0.384
Last cigarette before testing (h)	4.83	12.22	0.42–72	3.47	4.11	0.83–13.08	0.53	0.595
Years smoking	21.35	16.04	1–55	25.27	15.50	0.50–53	−0.94	0.349
Smoking days per week	6.78	0.68	4–7	6.56	1.16	42187.00	0.92	0.359
Previous quit attempts	3.91	3.88	0–16	3.40	3.42	0–15	0.53	0.597
Varenicline (%)	14 %			20 %			0.46	0.496
Bupropion (%)	0 %			8 %			3.06	0.080
Nicotine replacement (%)	32 %			20 %			1.16	0.282
Counseling (%)	11 %			24 %			1.92	0.166
Cigarettes per day baseline	14.95	7.11	2–30	17.68	9.38	2–40	−1.31	0.197
Cigarettes per day 1-month follow-up	4.89	6.40	0–22	0.52	1.53	0–6	3.93	0.000***
Cigarettes per day 2-month follow-up	6.78	6.52	0–22	0.46	1.56	0–6	5.58	0.000***
Cigarettes per day 3-month follow-up	8.86	6.73	0–25	0.00	0.00	n/a	8.02	0.000***

*FTND* Fagerström Test for Nicotine Dependence, *AUDIT* Alcohol Use Disorder Identification Test, *QSU* Questionnaire of Smoking Urges

^a^The number of relapsers and non-relapsers does not add up to the total number of participants including in the MPlus analyses because 12-week follow-up data was missing for a few participants. However, MPlus does include these participants in the analyses because missing data is handled using full information maximum likelihood

* *p* < .05; *** *p* < .001

### Questionnaires

Before EEG data acquisition, participants were asked to report basic information about their smoking behavior, including smoking days per week, number of cigarettes per day, years of smoking, number of previous quit attempts, and the number of hours since their last cigarette. Additionally, the Fagerström Test for Nicotine Dependence (FTND) was used to assess nicotine dependence levels (Heatherton et al. 1991; Vink et al. 2005). To assess subjective craving for a cigarette, participants completed the brief version of the Questionnaire of Smoking Urges (Cox et al. 2001). Alcohol use patterns were measured using the Alcohol Use Disorder Identification Test (AUDIT) (Saunders et al. 1993).

### Task paradigms

#### Smoking cue reactivity

Thirty-two smoking-related and 32 neutral pictures were presented to the participants. Participants were asked to passively view these pictures while paying attention to the content of the pictures. Smoking pictures display people smoking or handling cigarettes or smoking-related objects such as a package of cigarettes. Neutral pictures display people involved in similar scenes, however, without smoking or cigarettes or non-smoking-related objects. Smoking-related and neutral pictures were matched for number and gender of the persons displayed, type of activities, and number of objects. All pictures were presented twice resulting in 128 trials. Smoking-related and neutral pictures were presented in an event-related quasi-random order so that there were no successions of more than three pictures from the same category. Picture presentation lasted 1000 ms with an inter-stimulus interval randomly varying from 800 to 1200 ms, with an average of 1000 ms.

#### Go-NoGo task

Inhibitory control was measured in the context of neutral and smoking-related cues. The Go-NoGo task used for this purpose was identical to the one described by Luijten et al. (2011) except that we now measured inhibition of smoking-related and neutral cues in separate blocks (instead of in an random event-related design). Inhibitory control in the neutral context was measured first, followed by the smoking-related context. In order to create a neutral and smoking-related context, Go and NoGo stimuli consisted of neutral and smoking-related pictures with either a blue or yellow frame. Picture content was similar to the pictures described in the cue reactivity paradigm (all pictures in this task differed from the ones used in the cue reactivity task). Frame color indicated whether a stimulus was a Go or a NoGo trial. The attribution of the frame color to Go versus NoGo trials was counterbalanced across participants. Participants were instructed to respond to Go trials by pressing a button as fast as possible and to withhold their response in NoGo trials. One hundred twelve different smoking-related pictures and 112 neutral pictures were each presented four times during the task, once as a NoGo stimulus and three times as a Go stimulus. This means that 25 % of all trials were NoGo trials and that the proportion of smoking and non-smoking pictures in the task was equal (i.e., 112 NoGo trials per picture category and 336 Go trials per picture category). Each picture was presented for 200 ms and followed by a black screen for a random duration between 1020 and 1220 ms. Participants were given the opportunity to take a short break at four time moments during task performance.

#### Eriksen Flanker task

The Eriksen Flanker task was used to measure error processing (Eriksen and Eriksen 1974). The version in the current study is identical to the Eriksen Flanker task described by Marhe et al. (2013). Stimuli in this task consisted of four letter strings (i.e., SSSSS, HHHHH, HHSHH, SSHSS). Participants were asked to respond to the middle letter with a left or right button press for the S and H, respectively. The task consisted of 200 congruent (i.e., SSSSS, HHHHH) and 200 incongruent trials (i.e., SSHSS, HHSHH). Each trial started with a fixation cue (^) for 150 ms, followed by the letter string that was presented for 52 ms. The letter string was followed by a 648-ms blank response window. Thereafter, the participants were provided with feedback for 500 ms consisting of a “+,” “−,” or “!” symbol for correct, incorrect, or missed responses, respectively. The inter-trial interval consisted of a 100-ms blank screen.

### EEG recording and data reduction

EEG was recorded using the Biosemi Active-Two amplifier system (Biosemi, Amsterdam, the Netherlands) from 34 scalp sites (positioned following the 10–20 International System with two additional electrodes at FCz and CPz) with active Ag/AgCl electrodes mounted in an elastic cap. Six additional electrodes were attached to the left and right mastoids, to the two outer canthi of both eyes (HEOG), and to an infraorbital and a supraorbital region of the right eye (VEOG). All signals were digitalized with a sample rate of 512 Hz and 24-bit A/D conversion. During offline processing, no more than four bad channels per subject were removed and replaced by new values per channel calculated based on topographic interpolation. Data were re-referenced to computed linked mastoids. EEG and EOG activity was filtered with a bandpass of 0.15–30 Hz (24 dB/octave slope). After ocular correction (Gratton et al. 1983), epochs including an EEG signal exceeding ±100 μV were excluded from the average. The mean 100-ms pre-event period served as baseline. After baseline correction, average ERP waves were calculated for artifact-free trials at each scalp site for the different task conditions separately.

EEG data for the smoking cue reactivity paradigm was segmented in epochs of 2000 ms (200 ms before and 1800 ms after picture presentation). Based on visual inspection and in line with previous studies, the P300 and LPP were, respectively, defined as the mean amplitude within the 300–500- and 500–1000-ms time interval after picture presentation and studied at the midline electrodes Fz, Cz, and Pz (Littel et al. 2012; McDonough and Warren 2001; Warren and McDonough 1999). The mean number of analyzable segments for neutral and smoking-related pictures was 57.03 (range 25–65) and 57.23 (range 27–64), respectively.

EEG data for the Go-NoGo task was segmented in epochs of 1650 ms (200 ms before and 1450 ms after picture presentation). Segments with incorrect responses (miss for Go trials or false alarm for NoGo trials) were excluded from EEG analyses. The N2 was defined as the average amplitude within the 200–300-ms time interval after stimulus onset. Visual inspection revealed that the NoGo N2 amplitude was specifically larger compared to Go trials at parietal electrodes and was therefore studied at a cluster of central-parietal electrodes (Cz, CPz, CP1, CP2, and Pz). The P3 was defined as the average amplitude within the 300–600-ms time interval after stimulus onset and was studied at a cluster of central electrodes, including FCz, Cz, C3, C4, and CPz (Kiefer et al. 1998; Luijten et al. 2011). The mean number of analyzable NoGo epochs for smoking and neutral pictures was 74.86 (range 12–108) and 74.74 (range 21–105) for NoGo trials.

EEG data for the Eriksen Flanker task was segmented in epochs of 900 ms (200 ms before and 700 ms after responses). The ERN was defined as the mean amplitude within the 25–100-ms time interval after the response and was studied at the midline electrodes Fz, FCz, and Cz (Marhe et al. 2013). The Pe was defined as the mean amplitude within the 200–400-ms time interval after the response and was studied at central-parietal midline electrodes Cz, CPz, and Pz (Nieuwenhuis et al. 2001; Overbeek et al. 2005). The mean number of analyzable correct and incorrect segments was 347.45 (range 211–387) and 29.12 (range 7–135), respectively.

### Analyses

Analyses and results regarding the effects of basic task manipulations on behavior and ERPs are presented in the supplementary materials. All prediction models for smoking relapse and resumption were performed in MPlus 7. Seven-day point prevalence at 12-week follow-up was used as the primary outcome measure for smoking relapse (Wray et al. 2013). Nicotine dependence (i.e., FTND scores) and subjective craving at baseline were included as predictors in all analyses. Relapse risk in the current study was found to be substantially higher in females compared to males (Table 1); therefore, gender was also included as predictor in relapse models. The analyses for inhibitory control and error processing further included the primary behavioral measures for these processes, i.e., NoGo accuracy for inhibitory control averaged over smoking and neutral pictures and post-error slowing for error processing, which is calculated by subtracting reaction times for trials following correct trials from reaction times for trials following incorrect trials (Rabbitt 1966). Finally, all models included one composite score for the relevant ERP at hand (i.e., smoking minus neutral for the P300 and LPP, NoGo minus Go averaged over smoking and neutral pictures for the N2 and P3, and incorrect minus correct for the ERN and PE). Difference scores were averaged over the included electrodes. Six separate models were calculated, with each model including one ERP. We have two main reasons for running separate models for all ERPs, the first reason being the strong correlation between P300 and LPP and N2 and P3 amplitudes, *ρ =* 0.79, *p* < .001 and *ρ =* 0.50, *p* < .001, respectively. The second reason is the exploratory character of the current study because studies investigating the association between ERPs and smoking relapse and resumption are largely lacking. Parameters were estimated with maximum likelihood estimation with robust standard errors (MLR). The categorical nature of the relapse variable was handled with the CATEGORICAL ARE option within logistic regression analyses.

The number of smoked cigarettes per day at 4-, 8-, and 12-week follow-up was used as outcome measure for the prediction of smoking resumption. A latent growth curve approach using the MLR estimator (reported in *β*) was used to assess the increase of smoking behavior over time after the quit attempt. Latent growth models approach the analysis of repeated measures from the perspective of an individual growth curve for each subject; each growth curve has a certain initial level (intercept) and a certain rate of change over time (slope) (Duncan et al. 2006). All predictor variables in the latent growth curve models are identical to the logistic regression analyses, with the exception of gender, which is not included in latent growth curve models. In contrast to relapse rates, the number of smoked cigarettes per day at 4, 8, and 12 weeks did not differ between males and females, *t*(64) = −0.070, *p* = 0.944, *t*(60) = 0.47, *p* = 0.642, *t*(60) = 0.05, *p* = 0.962. For this reason, and because we aimed to include as few predictors as possible given the current sample size, gender was not included as covariate in the smoking resumption models. The fit of the models was assessed by the following fit indexes: *χ*^2^, comparative fit index (CFI), Tucker-Lewis index (TLI), and root-mean-square error of approximation (RMSEA). Except for RMSEA values (which are satisfactory if <0.08), goodness-of-fit values >0.90 are considered an acceptable fit (Bentler and Bonnet 1980). Missing data were handled by full information maximum likelihood (FIML).

## Results

### Smoking cue reactivity

**Fig. 1:**
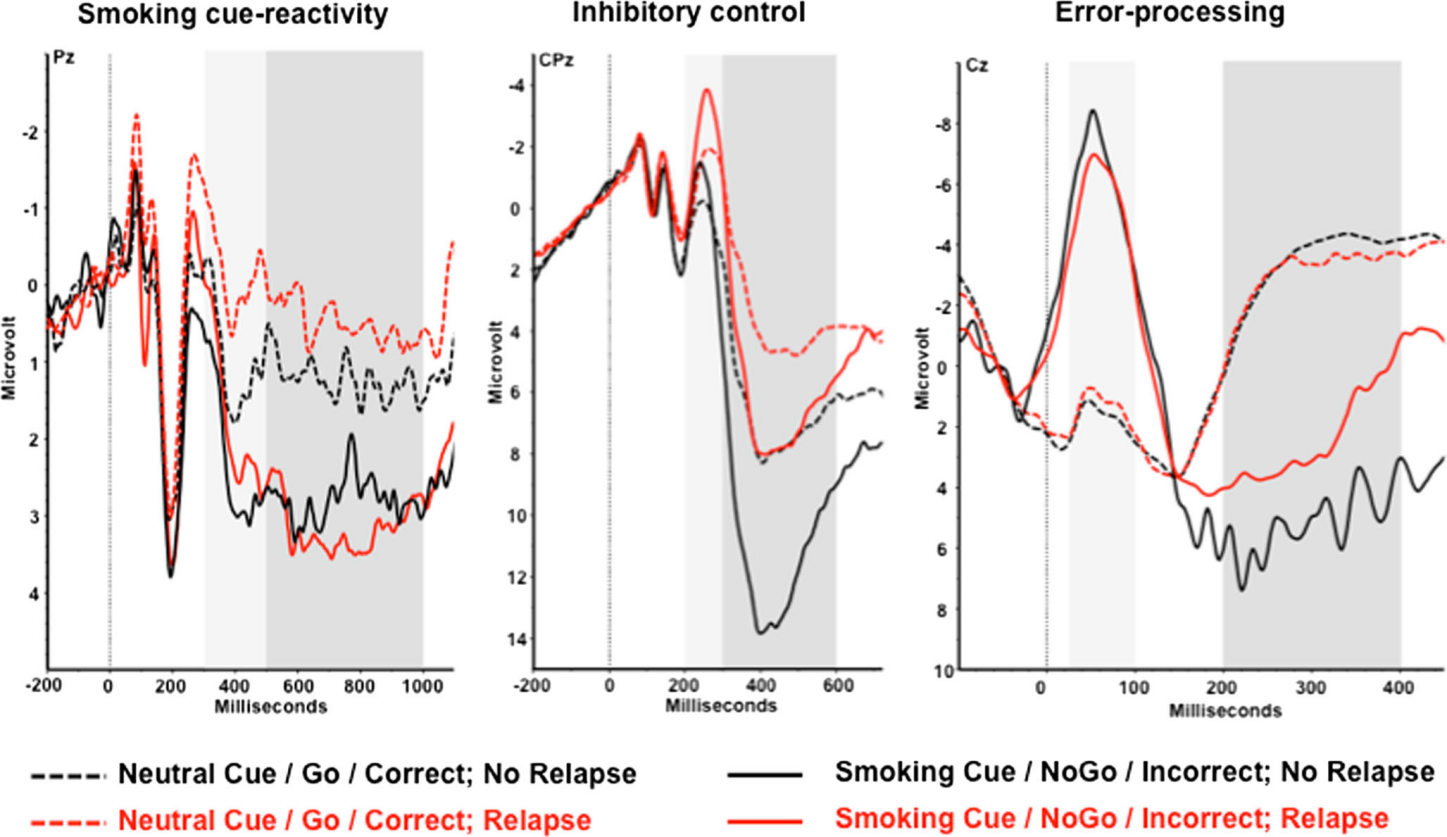
Event-related potentials reflecting smoking cue reactivity, inhibitory control, and error processing separate for smokers who relapsed and smokers who did not relapse. The *light grey area* for smoking cue reactivity represents the 300–500-ms time interval for the P300. The *dark grey area* represents the 500–1000-ms interval for the LPP. For inhibitory control, the *light* and *dark grey areas* represent the 200–300-ms interval for the N2 and the 300–600 ms for the P3, respectively. For error processing, the *light* and *dark grey areas* represent the 25–100-ms interval for the ERN and 200–400-ms interval for the Pe. Electrodes for visualization were selected so that the displayed electrode was included in the analyses of both ERPs for each cognitive process: Pz for smoking cue reactivity, CPz for inhibitory control, and Cz for error processing

ERP amplitudes are displayed in Fig. 1. Tables 2 and 3 show the outcomes of the logistic regressions and the latent growth curve models predicting smoking relapse and the increase in smoking behavior over time. The P300 and LPP reflecting smoking cue reactivity were predictive neither for relapse nor for the increase in smoking over time after the quit attempt. Gender predicted relapse in models including the P300 and LPP. The risk for relapse in women was higher than the relapse risk for men. Increased baseline nicotine dependence scores were found to be associated with an increase in smoking behavior over time.

**Table 2:** Logistic-regression outcomes

	OR	95 % CI	*p* value
P300 Cue reactivity model			
Nicotine dependence	1.03	0.81–1.31	0.791
Craving	1.00	0.95–1.04	0.844
Gender	0.28	0.10–0.77	0.013*
P300	1.06	0.85–1.32	0.615
LPP—cue reactivity model			
Nicotine dependence	1.04	0.82–1.32	0.750
Craving	1.00	0.95–1.05	0.874
Gender	0.29	0.11–0.78	0.015*
LPP	1.16	0.92–1.45	0.207
ERN—error-processing model			
Nicotine dependence	0.97	0.74–1.27	0.802
Craving	0.99	0.94–1.04	0.560
Gender	0.27	0.09–0.78	0.015*
Post-error slowing	0.99	0.96–1.01	0.211
ERN	1.04	0.91–1.19	0.537
Pe—error-processing model			
Nicotine dependence	0.99	0.76–1.28	0.917
Craving	0.98	0.93–1.03	0.440
Gender	0.24	0.08–0.72	0.011*
Post-error slowing	0.99	0.96–1.06	0.157
Pe	0.93	0.83–1.01	0.074
N2—inhibitory control model			
Nicotine dependence	1.02	0.77–1.31	0.866
Craving	1.00	0.95–1.05	0.903
Gender	0.24	0.08–0.68	0.007**
NoGo accuracy	0.97	0.94–1.01	0.124
N2	0.83	0.65–1.05	0.118
P3—inhibitory control model			
Nicotine dependence	0.98	0.75–1.28	0.907
Craving	1.00	0.95–1.05	0.889
Gender	0.17	0.05–0.52	0.002**
NoGo accuracy	0.97	0.93–1.01	0.097
P3	0.81	0.64–0.97	0.021*

*OR* Odds Ration, *CI* Confidence Interval

* *p* < .05; ** *p* < .01

**Table 3:** Latent growth curve modeling outcomes

	Fit-measures					Intercept						Slope					
	*χ* ^2^ (*df*)	*p*-*χ* ^2^	CFI	TLI	RMSEA	*β* value	SE	*p* value	RV^a^	SE-RV	*p*-RV	*β* value	SE	*p*	RV^a^	SE-RV	*p*-RV
Single growth curve model	0.15(2)	0.94	1	1.06	0.00	0.66	0.12	0.000***	20.55	7.37	0.005**	0.37	0.12	0.002**	7.95	3.58	0.026*
P300 cue reactivity model	1.82(5)	0.87	1	1.08	0.00				17.43	6.44	0.007**				6.13	2.24	0.006**
Nicotine dependence						0.11	0.11	0.308				0.25	0.13	0.049*			
Craving						0.21	0.15	0.150				−0.10	0.12	0.422			
P300						−0.16	0.10	0.118				0.00	0.11	0.981			
LPP—cue reactivity model	1.82(5)	0.87	1	1.08	0.00				17.79	6.66	0.008**				6.10	2.23	0.006**
Nicotine dependence						0.11	0.11	0.308				0.25	0.13	0.049*			
Craving						0.21	0.15	0.150				−0.10	0.12	0.422			
LPP						−0.16	0.10	0.118				0.00	0.11	0.981			
ERN— error-processing model	6.77(6)	0.34	0.99	0.98	0.05				17.13	6.95	0.014*				5.61	1.95	0.004**
Nicotine dependence						0.23	0.13	0.091				0.17	0.15	0.264			
Craving						0.22	0.15	0.164				−0.18	0.12	0.127			
Post-error slowing						0.06	0.11	0.603				−0.21	0.11	0.048*			
ERN						−0.17	0.13	0.208				0.26	1.73	0.084			
Pe—error-processing model	2.24(6)	0.90	1	1.1	0.00				18.10	7.26	0.013*				6.16	2.20	0.005**
Nicotine dependence						0.15	0.12	0.205				0.28	0.11	0.015*			
Craving						0.21	0.16	0.172				−0.19	0.12	0.124			
Post-error slowing						0.09	0.11	0.445				−0.25	0.11	0.029*			
Pe						−0.05	0.12	0.676				−0.04	0.13	0.778			
N2—inhibitory control model	0.99(6)	0.99	1	1.15	0.00				18.68	6.58	0.005**				5.81	2.19	0.008**
Nicotine dependence						0.19	0.11	0.089				0.19	0.12	0.109			
Craving						0.15	0.15	0.344				−0.04	0.12	0.754			
NoGo accuracy						−0.16	0.16	0.323				−0.10	0.12	0.410			
N2						−0.04	0.15	0.781				−0.13	0.13	0.341			
P3—inhibitory control model	3.71(6)	0.72	1	1.06	0.00				18.37	6.53	0.005**				5.46	2.01	0.007**
Nicotine dependence						0.2	0.12	0.100				0.14	0.12	0.230			
Craving						0.15	0.15	0.317				−0.04	0.12	0.747			
NoGo accuracy						−0.16	0.16	0.315				−0.10	0.12	0.411			
P3						0.01	0.12	0.958				−0.25	0.13	0.054			

*CFI* Comparative Fit Index, *TLI* Tucker-Lewis Index, *RMSEA* root-mean-square error of approximation, *RV* residual variance, *SE* standard error

^a^Unstandardized versions of residual variances (RV) are reported. All other presented measures are standardized

* *p* < .05; ** *p* < .01; *** *p* < .001

### Inhibitory control

Consistent with the previous relapse models, women were found to have an increased chance of relapse compared to men in both logistic regression models including the N2 and P3. Furthermore, as can be observed in Table 2, smaller P3 amplitudes were found to be associated with an increased risk for relapse as well as a trend for increased smoking behavior over time.

### Error processing

The models relating error processing indices to relapse again showed that women were more likely to relapse than men. Additionally, a trend (i.e., *p* = 0.074) was observed for the Pe, indicating that smaller Pe amplitudes may be associated with an increased chance of smoking relapse. Both latent growth curve models including the ERN and Pe showed that less pronounced post-error slowing is related to an increase in smoking behavior over time. Additionally, a trend (i.e., *p* = 0.084) was observed for the ERN, indicating that smaller ERN amplitudes may be associated with increased smoking behavior over time. Finally, higher baseline nicotine dependence was found to be associated with increased smoking behavior over time in the latent growth curve model including the Pe.

## Discussion

The link between smoking cessation and ERPs reflecting main cognitive processes involved in addictive behaviors was investigated in the current study. Results showed that reduced P3 amplitudes associated with inhibitory control are related to increased risk of relapse. Results further showed that reduced post-error slowing, the main behavioral measure reflecting error processing, is associated with an increase in smoking over time after a quit attempt. In contrast to our hypotheses, ERPs reflecting smoking cue reactivity are not associated with smoking relapse or resumption in the current study.

Previous research has shown that smokers are characterized by reduced inhibitory control (for meta-analysis, see Smith et al. 2014). The current results add to this knowledge that reduced P3 amplitudes, reflecting the urgent inhibitory brake in the pre-motor cortex (Luijten et al. 2014), seem to be associated with lower chances of successful smoking cessation. The current finding that P3 amplitudes, rather than N2 amplitudes, are associated with smoking relapse indicates that impaired urgent motor inhibition processes (Kok et al. 2004) rather than impaired conflict detection (Enriquez-Geppert et al. 2010; Nieuwenhuis et al. 2003) are related to difficulties with smoking cessation. This finding is in line with earlier research showing that reduced brain activation in the pre-supplementary motor area is associated with a stronger link between craving and smoking after a quit attempt (Berkman et al. 2011). Furthermore, previous research has shown that inhibitory control does not improve after extended abstinence (Bradstreet et al. 2014; Dawkins et al. 2009). This further emphasizes that poor inhibitory control may be a long-term risk factor for smoking relapse and therefore should be targeted in smoking cessation interventions. Recent findings in overweight people (Veling et al. 2014) as well as in heavy drinkers (Houben et al. 2012; Jones and Field 2013) suggest that inhibitory control training could be an effective tool to reduce risky addictive behaviors.

In line with previous studies linking reduced error processing to increased cocaine use at 3-month follow-up (Luo et al. 2013; Marhe et al. 2013), we found that reduced post-error slowing is related to an increase in smoking behavior over time after a quit attempt. Conceptually, post-error slowing reflects a post-error compensatory process that is associated with increased post-error accuracy, suggesting that post-error slowing represents a performance marker of monitoring of ongoing behavior (Hajcak et al. 2003). The current link between smoking resumption and post-error slowing suggests that difficulties with behavioral monitoring are associated with an increase in smoking after a quit attempt. However, given that both the Pe and ERN were associated with smoking relapse and resumption at trend levels only, more research is needed in a larger sample to be able to formulate clear conclusions about the association between error processing and smoking cessation. Notably, error processing-related analyses in the current study were performed with less power than the smoking cue reactivity and inhibitory control analyses, because some participants did not make enough errors during the Eriksen Flanker task to calculate reliable ERN and Pe amplitudes.

P300 and LPP amplitudes reflecting enhanced attentional processing of smoking cues were not related to smoking relapse and resumption in the current study. Given that smokers show larger P300 and LPP amplitudes for smoking cues relative to neutral cues in the current, as well as in previous, studies (Littel et al. 2012), we expected that increased attentional processing of smoking cues in real life would trigger craving and would therefore interfere with smoking cessation. The discrepancy between the findings of the current study and studies showing that enhanced cognitive processing of smoking is related to reduced quit success (Janes et al. 2010; Powell et al. 2010; Versace et al. 2014; Waters et al. 2003b) may be explained by the timing of the measurement of smoking cue reactivity. In the current study, participants were tested in the week before the quit attempt and were abstinent for only 1 h before testing. Smoking cue reactivity in several brain regions including the striatum seems to be potentiated after 24-h of smoking abstinence (McClernon et al. 2009), suggesting that testing during the initial stages of smoking cessation would result in amplified cue reactivity that has a stronger relation with smoking cessation. Indeed, another study that did not find the association between smoking cue reactivity and smoking cessation also measured smoking cue reactivity in the preceding 2 weeks before the quit attempt (Waters et al. 2003a), while some studies with positive results tested participants during the first hours of the quit attempt (Powell et al. 2010; Waters et al. 2003b). Another explanation for the negative findings regarding cue reactivity may be the heterogeneity of the sample of smokers in our study. While most previous studies included only heavy smokers (i.e., at least ten of more cigarettes a day), there was no requirement about the number of cigarettes smoked per day in the current study. While a heterogeneous sample has the advantage that results can be generalized to a larger population of smokers, the disadvantage is that different smoking cessation mechanisms may be involved for different types of smokers (Shiffman et al. 1995), and this can hamper the finding of more general patterns.

As the current study measured both cognitive control-related brain activation (i.e., inhibitory control and error processing) and brain reactivity to conditioned smoking cues, the current findings can be interpreted in the context of dual-process models of addiction. Dual-process models suggest that decreased cognitive control over increased motivation to consume substances of abuse is the core mechanism involved in the continuation of addictive behavior (Field and Cox 2008; Volkow et al. 2004; Wiers et al. 2007). In light of these dual-process models, findings of the current study suggest that reduced cognitive control may be a more promising candidate to predict smoking relapse before the actual quit attempt compared to enhanced motivational properties of conditioned substance cues.

A few methodological issues deserve special attention in the interpretation of the current results. First, N2 amplitudes in the inhibitory control task in the current study showed a rather central-parietal distribution, while N2 peaks in inhibitory control tasks usually have a frontocentral distribution (Kaiser et al. 2006; Kok et al. 2004). The main difference between the current study and traditional studies measuring inhibitory control-related ERPs is the use of pictorial stimuli in addition to the Go and NoGo cues that consisted of a blue or yellow border around the pictures. The pictures included in the current task design showed either neutral or smoking-related cues. Given that the processing of more complex stimuli in general and addiction-related cues specifically is known to be a mainly parietal process (Littel et al. 2012), the addition of pictorial stimuli may have caused a shift in the distribution of N2 peaks from frontocentral to a central-parietal site.

Second, follow-up measures in the current study were limited in time to 3 months after the quit date. While most smokers relapse within the first few weeks after their quit attempt, relapse into smoking still occurs after extended time periods (Stapleton 1998). As mechanisms of smoking relapse may differ for early and late relapsers, it would be interesting for future studies to include follow-up measures up to 12 months after the quit attempt.

Third, relapse risk in the current study was found to be substantially higher in females compared to males. This finding is in contrast to the overall literature. A pooled analysis of studies investigating predictors of quit success in the general adult population did not find a gender effect on relapse (Vangeli et al. 2011). Our strategy to include gender in the relapse analyses was therefore based on the assumption that the current finding of increased relapse risk in females is not representative for the overall smoking population. By including gender in the analyses regarding relapse, we accounted for the variance in relapse rates related to gender in the current study specifically.

Fourth, while the inclusion of three cognitive tasks in the current study is a strength, it also resulted in multiple analyses per outcome variable. This relatively large number of analyses is a limitation of the current study, as it increased the chances for false-positive findings. However, given that the current study is the first using this type of design and analyses techniques to investigate the association between ERPs reflecting multiple cognitive processes related to addictive behaviors and smoking relapse and resumption, we think that the rather explorative nature of the analyses is justified. Nevertheless, replication of the current findings is strongly warranted.

Fifth, the current sample of smokers is also rather heterogeneous in terms of types of support that were used during the quit attempt. Such a naturalistic sample of smokers has advantages in terms of generalizability, yet it is also a limitation given that we cannot control for the effects of different types of treatment during the quite attempt because of limited power. However, we did perform additional analyses including a single treatment factor (additional support versus no additional support), which did not change the main conclusion of the analyses (for more details, see supplementary materials).

Finally, while neurocognitive measures may be able to predict smoking relapse and can potentially contribute to individualized smoking cessation support, one of the biggest challenges in this research field is moving from group-level associations with smoking cessation to individual prediction of smoking cessation (Marhe et al. 2014). EEG measures are quite promising for this purpose, as they have an adequate internal reliability (Olvet and Hajcak 2009; Wostmann et al. 2013) and are less expensive and easier to implement in clinical practice compared to fMRI (Marhe et al. 2014; Versace et al. 2012).

## Conclusions

The current study is the first to investigate the association between ERPs reflecting smoking cue reactivity, inhibitory control, and error processing in relation to smoking relapse and resumption. Findings revealed that smaller P3 amplitudes associated with inhibitory control-related brain activation predicted increased relapse risks. Furthermore, behavioral- and brain-related measures of error processing may be promising in the prediction of smoking cessation, while ERPs reflecting smoking cue reactivity were unrelated to smoking cessation in the current study. These findings suggest that strategies to increase cognitive control in smokers are worth further investigation, as this may contribute to the development of more effective smoking cessation treatments.

## Electronic supplementary material

Below is the link to the electronic supplementary material.

ESM 1 (DOC 170 kb)
