# Supplementary material for: Event-related potentials reflecting smoking cue reactivity and cognitive control as predictors of smoking relapse and resumption
Source: Psychopharmacology (Berl). 2016 Jun 8;233:2857–68. doi: 10.1007/s00213-016-4332-8 (PMC4933734; doi:10.1007/s00213-016-4332-8)
Supplement: Supplementary file 1 — (DOC 170 kb) [file 213_2016_4332_MOESM1_ESM.doc]

Online supplementary materials *Psychopharmacology* for

**Event-related potentials reflecting smoking cue-reactivity and cognitive control as predictors of smoking relapse and resumption**

Maartje Luijten, PhD^ab*^, Marloes Kleinjan, PhD^c^, Ingmar. H.A. Franken, PhD^b^

^a^Behavioural Science Institute, Radboud University, P.O. Box 9104, 6500 HE, Nijmegen, the Netherlands
^b^Institute of Psychology, Erasmus University Rotterdam, P.O. Box 1738, 3000 DR, Rotterdam, the Netherlands

^c^Trimbos Institute (Netherlands Institute of Mental Health and Addiction), P.O. Box 725, 3500 AS Utrecht, The Netherlands

**^*^Correspondence**

Maartje Luijten, PhD

Email: [m.luijten@bsi.ru.nl](mailto:m.luijten@bsi.ru.nl)

**1. Analyses**

We report these additional analyses in order to demonstrate the effects of task manipulations on behaviour and Event-Related Potentials (ERPs). First, paired samples *t*-tests and Repeated Measures ANOVA’s (RM-ANOVA) with Greenhouse-Geisser adjusted *p*-values were performed in order to check task manipulation effects on both behavioral and ERPs measures. To investigate whether smokers showed enlarged P300 and LPP amplitudes for smoking relative to neutral pictures two Picture (Smoking versus Neutral) x Electrode (Fz, Cz, Pz) RM-ANOVA’s were performed for the P300 and LPP and separately. A Picture (smoking versus neutral) x Inhibition (Go versus NoGo trails) RM-ANOVA was performed for accuracy rates during the Go-NoGo task. To establish whether Inhibition condition and Picture type influenced N2 and P3 amplitudes, two Picture x Inhibition x Electrode (Cz, CPz, CP1, CP2 and Pz for the N2 and FCz, Cz, C3, C4 and CPz for the P3) RM-ANOVA’s were performed. No interaction-effects and follow-up tests are reported for Electrode effects. The design of the current Eriksen Flanker task resulted in the following factors of interest: a) Congruency (incongruent versus congruent trials), b) Correctness (correct versus incorrect trials), and c) Post-correctness (post-correct versus post-error trials). Paired samples *t*-tests were performed to investigate the effect of Congruency on accuracy rates and the effect of all three factors on reaction times. To asses whether the ERN and Pe were enhanced for incorrect versus correct trials two Correctness x Electrodes (Fz, FCz, Pz for the ERN and Cz, CPz and Pz for the Pe) RM-ANOVA’s were performed for the ERN and Pe separately.

To investigate the influence of treatment support during the quit attempt on our findings regarding the association between ERPs and smoking relapse and resumption we performed all logistic regression analyses and growth-curve models again while including an additional Treatment factor. This treatment factor was a dichotomous variable representing whether or not someone used any type of additional support during the quit attempt.

**2. Results**

2.1 Smoking cue reactivity

Both the P300 and LPP showed a significant effect of Picture, *F*(1,65) = 29.43, *p*<.001 and F(1,65) = 47.80, *p*<.001 respectively, thereby confirming that the current smokers have enlarged amplitudes for smoking relative to neutral pictures. Main effects of Electrodes were significant for both the P300 and LPP, *F*(2,64)= 138.77, *p*<.001 and *F*(2,64)=62.81, p<.001 respectively.

2.2 Inhibitory control

Means and standard deviations for accuracy rates and reaction times are displayed in Table S1. A main effect of Inhibition showed that participants were less accurate for NoGo trials compared to Go trials, *F*(1,65)=151.44, *p*<.001. No main or interaction effect of Picture was found for Accuracy rates. ERP amplitudes for the N2 and P3 showed the same pattern. The main effect of Inhibition was significant for both the N2 and P3, *F*(1,65)=10.28, *p*=.002 and *F*(1,65)=70.80, *p*<.001 respectively, indicating that both N2 and P3 amplitudes were larger for NoGo than Go trials. Main effects of Electrodes were significant for both the N2 and P3, *F*(4,62)= 28.33, *p*<.001 and *F*(4,62)=11.24, *p*<.001, respectively. Main effects of Picture or Condition x Picture interactions were neither significant for the N2 nor the P3. Given the overall lack of a picture effect on both behavior and ERPs, the predictors representing inhibitory control in the analyses regarding smoking cessation were averaged over smoking and neutral pictures.

2.3 Task performance and ERPs

See Table S2 for means and standard deviations for accuracy rates and reaction times. In line with the expected task manipulations it was found that accuracy rates were lower for incongruent compared to congruent trials *t*(59)=-10.34, *p*<.001. Additionally, reaction times are longer for incongruent versus congruent trials *t*(59)=22.63, *p*<.001 and also longer for post-error versus post-correct trials, *t*(59)=5.99, *p*<.001, indicating that participants showed post-error slowing, which is an important behavioral indication for error-processing. A significant effect for Correctness was found for both the ERN and the Pe, *F*(1,59) = 89.57, *p*<.001 and F(1,59) = 59.69, *p*<.001 respectively, showing that the current smokers process erroneous responses more strongly than correct responses. Main effects of Electrodes were significant for both the ERN and Pe, *F*(2,58)= 9.68, *p*=.001 and *F*(2,58)=4.49, *p*=.024 respectively. Both the ERN and Pe showed significant Correctness x Electrodes interaction effects, *F*(2,58)= 67.88, *p*=.001 and *F*(2,58)=17.55, *p*<.001 respectively.

2.4 Treatment factor

Overall, adding the Treatment factor did not change the results regarding the association between ERPs and smoking relapse and resumption. The Treatment factor was not significant in any of the logistic regressions predicting relapse and adding the Treatment factor did not change the significance of any of the other factors in the logistic analyses. See table S3 for an overview of the results of all logistic models including the Treatment factor.

In the growth-curve regarding smoking resumption the Treatment factor showed a significant effect on the intercept in 3 out of the 5 analyses. This means that generally the participants who used any form of treatment smoked more after the quit attempt. The association between the intercept and any of the other factors, including the ERPs, did not change after adding the Treatment factor. The treatment effect also did not influence the increase in smoker over time as no effect of Treatment was found for the slope of the number of cigarettes over time. In the analyses predicting the slope of the increase in smoking over time, baseline nicotine dependence levels were no longer significant in predicting the slope. This may be explained by the fact that participants with higher nicotine dependence levels more often used treatment (FTND scores at baseline differ significantly between participants who used additional treatment and those who did not, *t*(65)=3.41, *p*=.001). The effect of post-error slowing changed from significance into a trend (*p*=.056) in the analysis including the ERN, however, post-error slowing remained significant in the analysis including the Pe. The significance of all other factors included in the model to predict the increase of smoking over time did not change. See table S4 for an overview of the results of all growth-curve models including the Treatment factor.

Table S1. Behavioral outcomes Go-NoGo task

|  | Relapsers (N=36)* | | Non-relapsers (N=25)* | |  |  |
| --- | --- | --- | --- | --- | --- | --- |
|  | *Mean* | *SD* | *Mean* | *SD* | *t* | *p* |
| Go Neutral - % correct | 97.77 | 2.99 | 98.63 | 1.80 | -1.28 | 0.205 |
| Go Neutral - RT | 300.33 | 55.58 | 295.32 | 53.06 | 0.35 | 0.725 |
| NoGo Neutral - % correct | 76.09 | 14.38 | 79.36 | 14.87 | -0.86 | 0.393 |
| NoGo Neutral - RT incorrect trials | 270.35 | 65.57 | 263.38 | 70.12 | 0.40 | 0.693 |
| Go Smoking - % correct | 96.69 | 4.18 | 98.69 | 1.71 | **-2.26** | **0.028** |
| Go Smoking - RT | 311.05 | 56.44 | 307.96 | 62.65 | 0.20 | 0.842 |
| NoGo Smoking - % correct | 75.40 | 15.44 | 80.64 | 14.73 | -1.33 | 0.189 |
| NoGo Smoking - RT incorrect trials | 286.96 | 64.67 | 285.05 | 85.68 | 0.10 | 0.921 |

* The number of relapsers and non-relapsers does not add up to the total number of participants including in the MPlus analyses because 12 weeks follow-up data was missing for a few participants. However, Mplus does include these participants in the analyses because missing data is handled using full information maximum likelihood. RT: Reaction times

Table S2. Behavioral outcomes Eriksen Flanker task

|  | Relapsers (N=35)* | | Non-relapsers (N=22)* | |  |  |
| --- | --- | --- | --- | --- | --- | --- |
|  | *Mean* | *SD* | *Mean* | *SD* | *t* | *p* |
| Congruent - % correct | 91.21 | 10.30 | 92.30 | 9.08 | -0.40 | 0.688 |
| Congruent - % incorrect | 4.04 | 4.99 | 2.57 | 3.96 | 1.17 | 0.246 |
| Congruent - % miss | 4.74 | 7.46 | 5.14 | 7.88 | -0.19 | 0.850 |
| Congruent - RT correct | 445.25 | 41.66 | 439.70 | 49.69 | 0.45 | 0.651 |
| Incongruent - % correct | 81.17 | 12.33 | 83.59 | 12.60 | -0.72 | 0.477 |
| Incongruent - % incorrect | 11.49 | 8.40 | 9.77 | 9.55 | 0.71 | 0.480 |
| Incongruent - % miss | 7.34 | 8.91 | 6.64 | 9.21 | 0.29 | 0.775 |
| Incongruent - RT correct | 490.00 | 40.82 | 488.88 | 56.66 | 0.09 | 0.931 |
| Post - Correct RT | 470.21 | 39.88 | 464.91 | 48.56 | 0.45 | 0.655 |
| Post - Error RT | 485.93 | 43.46 | 486.86 | 56.37 | -0.07 | 0.945 |
| Post - error slowing - RT | 15.72 | 26.21 | 21.95 | 20.44 | -0.95 | 0.348 |

* The number of relapsers and non-relapsers does not add up to the total number of participants including in the MPlus analyses because 12 weeks follow-up data was missing for a few participants. However, Mplus does include these participants in the analyses because missing data is handled using full information maximum likelihood. RT: Reaction times.

Table S3 Latent-Growth Curve modelling outcomes

|  | OR | 95% CI | *p* |
| --- | --- | --- | --- |
| *P300 Cue-reactivity model* |  |  |  |
| Nicotine Dependence | 1.00 | 0.78-1.29 | 0.988 |
| Craving | 0.99 | 0.95-1.04 | 0.808 |
| Gender | **0.29** | **0.10-0.79** | **0.016** |
| Treatment | 1.49 | 0.48-4.66 | 0.492 |
| P300 | 1.07 | 0.85-1.34 | 0.575 |
| *LPP - Cue-reactivity model* |  |  |  |
| Nicotine Dependence | 1.01 | 0.78-1.31 | 0.937 |
| Craving | 1.00 | 0.95-1.04 | 0.833 |
| Gender | **0.29** | **0.11-0.80** | **0.016** |
| Treatment | 1.42 | 0.46-4.45 | 0.545 |
| P300 | 1.15 | 0.92-1.46 | 0.227 |
| *ERN - Error-processing model* |  |  |  |
| Nicotine Dependence | 0.94 | 0.70-1.27 | 0.699 |
| Craving | 0.99 | 0.94-1.04 | 0.555 |
| Gender | **0.27** | **0.10-0.79** | **0.016** |
| Treatment | 1.31 | 0.38-4.52 | 0.673 |
| Post-error slowing | 0.99 | 0.97-1.01 | 0.254 |
| ERN | 1.04 | 0.91-1.20 | 0.536 |
| *Pe - Error-processing model* |  |  |  |
| Nicotine Dependence | 0.97 | 0.74-1.28 | 0.839 |
| Craving | 0.98 | 0.93-1.03 | 0.440 |
| Gender | **0.25** | **0.08-0.73** | **0.011** |
| Treatment | 1.20 | 0.35-4.11 | 0.772 |
| Post-error slowing | 0.99 | 0.96-1.01 | 0.178 |
| Pe | 0.93 | 0.86-1.01 | 0.075 |
| *N2 - Inhibitory control model* |  |  |  |
| Nicotine Dependence | 1.00 | 0.76-1.30 | 0.974 |
| Craving | 1.00 | 0.95-1.05 | 0.839 |
| Gender | **0.24** | **0.08-0.70** | **0.009** |
| Treatment | 1.44 | 0.44-4.74 | 0.552 |
| NoGo Accuracy | 0.97 | 0.93-1.01 | 0.127 |
| N2 | 0.82 | 0.64-1.04 | 0.105 |
| *P3 - Inhibitory control model* |  |  |  |
| Nicotine Dependence | 0.96 | 0.73-1.27 | 0.785 |
| Craving | 0.99 | 0.94-1.05 | 0.836 |
| Gender | **0.17** | **0.05-0.53** | **0.003** |
| Treatment | 1.38 | 0.42-4.54 | 0.602 |
| NoGo Accuracy | 0.97 | 0.93-1.01 | 0.096 |
| P3 | **0.81** | **0.67-0.97** | **0.021** |

Table S4 Latent-Growth Curve modelling outcomes

|  | Fit-measures | | |  | |  | |  | | Intercept | |  | |  | |  | |  | |  | | Slope | |  | |  | |  |  | | |  |
| --- | --- | --- | --- | --- | --- | --- | --- | --- | --- | --- | --- | --- | --- | --- | --- | --- | --- | --- | --- | --- | --- | --- | --- | --- | --- | --- | --- | --- | --- | --- | --- | --- |
|  | *χ2* (df) | *p - χ2* | CFI | | TLI | | RMSEA | | *β* - value | | SE | | *p* | | RV* | | SE - RV | | *p* - RV | | *β* - value | | SE | | *p* | | RV* | | | SE - RV | p - RV | |
| Single Growth-Curve model | 0.15(2) | 0.94 | 1.00 | | 1.06 | | 0.00 | | **0.66** | | **0.12** | | **0.000** | | 20.55 | | 7.37 | | 0.005 | | **0.37** | | **0.12** | | **0.002** | | 7.95 | | | 3.58 | 0.026 | |
|  |  |  |  | |  | |  | |  | |  | |  | |  | |  | |  | |  | |  | |  | |  | | |  |  | |
| *P300 Cue-reactivity model* | 1.90(6) | 0.93 | 1.00 | | 1.10 | | 0.00 | |  | |  | |  | | 16.36 | | 6.44 | | 0.011 | |  | |  | |  | | 6.09 | | | 2.21 | 0.006 | |
| Nicotine Dependence |  |  |  | |  | |  | | 0.02 | | 0.12 | | 0.835 | |  | |  | |  | | 0.22 | | 0.14 | | 0.121 | |  | | |  |  | |
| Craving |  |  |  | |  | |  | | 0.19 | | 0.15 | | 0.190 | |  | |  | |  | | -0.10 | | 0.12 | | 0.385 | |  | | |  |  | |
| Treatment |  |  |  | |  | |  | | 0.26 | | 0.14 | | 0.060 | |  | |  | |  | | 0.09 | | 0.15 | | 0.550 | |  | | |  |  | |
| P300 |  |  |  | |  | |  | | -0.14 | | 0.10 | | 0.170 | |  | |  | |  | | 0.00 | | 0.11 | | 0.970 | |  | | |  |  | |
| *LPP - Cue-reactivity model* | 2.07(6) | 0.91 | 1.00 | | 1.09 | | 0.00 | |  | |  | |  | | 16.62 | | 6.58 | | 0.012 | |  | |  | |  | | 6.06 | | | 2.20 | 0.006 | |
| Nicotine Dependence |  |  |  | |  | |  | | 0.05 | | 0.11 | | 0.680 | |  | |  | |  | | 0.22 | | 0.13 | | 0.104 | |  | | |  |  | |
| Craving |  |  |  | |  | |  | | 0.18 | | 0.15 | | 0.216 | |  | |  | |  | | -0.11 | | 0.12 | | 0.377 | |  | | |  |  | |
| Treatment |  |  |  | |  | |  | | **0.27** | | **0.14** | | **0.047** | |  | |  | |  | | 0.09 | | 0.15 | | 0.541 | |  | | |  |  | |
| LPP |  |  |  | |  | |  | | -0.02 | | 0.10 | | 0.814 | |  | |  | |  | | 0.01 | | 0.12 | | 0.942 | |  | | |  |  | |
| *ERN - Error-processing model* | 7.10(7) | 0.42 | 1.00 | | 1.00 | | 0.02 | |  | |  | |  | | 16.21 | | 6.80 | | 0.017 | |  | |  | |  | | 5.60 | | | 1.94 | 0.004 | |
| Nicotine Dependence |  |  |  | |  | |  | | 0.13 | | 0.14 | | 0.356 | |  | |  | |  | | 0.15 | | 0.17 | | 0.372 | |  | | |  |  | |
| Craving |  |  |  | |  | |  | | 0.21 | | 0.16 | | 0.176 | |  | |  | |  | | -0.18 | | 0.12 | | 0.126 | |  | | |  |  | |
| Treatment |  |  |  | |  | |  | | 0.25 | | 0.14 | | 0.084 | |  | |  | |  | | 0.06 | | 0.15 | | 0.709 | |  | | |  |  | |
| Post-error slowing |  |  |  | |  | |  | | 0.09 | | 0.11 | | 0.408 | |  | |  | |  | | -0.20 | | 0.11 | | 0.056 | |  | | |  |  | |
| ERN |  |  |  | |  | |  | | -0.15 | | 0.14 | | 0.281 | |  | |  | |  | | 0.26 | | 0.15 | | 0.074 | |  | | |  |  | |
| *Pe - Error-processing model* | 2.34(7) | 0.94 | 1.00 | | 1.12 | | 0.00 | |  | |  | |  | | 17.14 | | 7.10 | | 0.016 | |  | |  | |  | | 6.16 | | | 2.19 | 0.005 | |
| Nicotine Dependence |  |  |  | |  | |  | | 0.06 | | 0.12 | | 0.632 | |  | |  | |  | | 0.26 | | 0.14 | | 0.057 | |  | | |  |  | |
| Craving |  |  |  | |  | |  | | 0.21 | | 0.16 | | 0.182 | |  | |  | |  | | -0.19 | | 0.12 | | 0.124 | |  | | |  |  | |
| Treatment |  |  |  | |  | |  | | 0.25 | | 0.15 | | 0.094 | |  | |  | |  | | 0.04 | | 0.16 | | 0.801 | |  | | |  |  | |
| Post-error slowing |  |  |  | |  | |  | | 0.12 | | 0.16 | | 0.301 | |  | |  | |  | | **-0.24** | | **0.11** | | **0.028** | |  | | |  |  | |
| Pe |  |  |  | |  | |  | | -0.30 | | 0.13 | | 0.807 | |  | |  | |  | | -0.03 | | 0.13 | | 0.805 | |  | | |  |  | |
| *N2 - Inhibitory control model* | 1.07(7) | 0.99 | 1.00 | | 1.17 | | 0.00 | |  | |  | |  | | 17.00 | | 6.41 | | 0.008 | |  | |  | |  | | 5.78 | | | 2.17 | 0.008 | |
| Nicotine Dependence |  |  |  | |  | |  | | 0.09 | | 0.11 | | 0.413 | |  | |  | |  | | 0.17 | | 0.14 | | 0.209 | |  | | |  |  | |
| Craving |  |  |  | |  | |  | | 0.12 | | 0.16 | | 0.448 | |  | |  | |  | | -0.40 | | 0.12 | | 0.724 | |  | | |  |  | |
| Treatment |  |  |  | |  | |  | | **0.31** | | **0.13** | | **0.018** | |  | |  | |  | | 0.04 | | 0.15 | | 0.798 | |  | | |  |  | |
| NoGo Accuracy |  |  |  | |  | |  | | -0.17 | | 0.15 | | 0.259 | |  | |  | |  | | -0.10 | | 0.12 | | 0.389 | |  | | |  |  | |
| N2 |  |  |  | |  | |  | | -0.07 | | 0.15 | | 0.634 | |  | |  | |  | | -0.13 | | 0.13 | | 0.317 | |  | | |  |  | |
| *P3 - Inhibitory control model* | 3.80(7) | 0.80 | 1.00 | | 1.08 | | 0.00 | |  | |  | |  | | 16.80 | | 6.38 | | 0.008 | |  | |  | |  | | 5.45 | | | 1.99 | 0.006 | |
| Nicotine Dependence |  |  |  | |  | |  | | 0.09 | | 0.11 | | 0.433 | |  | |  | |  | | 0.13 | | 0.14 | | 0.359 | |  | | |  |  | |
| Craving |  |  |  | |  | |  | | 0.13 | | 0.16 | | 0.403 | |  | |  | |  | | -0.04 | | 0.12 | | 0.723 | |  | | |  |  | |
| Treatment |  |  |  | |  | |  | | **0.30** | | **0.13** | | **0.024** | |  | |  | |  | | 0.04 | | 0.15 | | 0.811 | |  | | |  |  | |
| NoGo Accuracy |  |  |  | |  | |  | | -0.18 | | 0.16 | | 0.252 | |  | |  | |  | | -0.10 | | 0.12 | | 0.388 | |  | | |  |  | |
| P3 |  |  |  | |  | |  | | -0.01 | | 0.12 | | 0.944 | |  | |  | |  | | **-0.25** | | **0.13** | | **0.050** | |  | | |  |  | |

CFI; Comparative Fit Index, TLI; Tucker-Lewis Index, RMSEA; Root-Mean-Square Error of Approximation.
* Unstandardized versions of residual variances (RV) are reported. All other presented measures are standardized.
